# Dynamic evolution of the alpha (α) and beta (β) keratins has accompanied integument diversification and the adaptation of birds into novel lifestyles

**DOI:** 10.1186/s12862-014-0249-1

**Published:** 2014-12-12

**Authors:** Matthew J Greenwold, Weier Bao, Erich D Jarvis, Haofu Hu, Cai Li, M Thomas P Gilbert, Guojie Zhang, Roger H Sawyer

**Affiliations:** Department of Biological Sciences, University of South Carolina, Columbia, South Carolina USA; Department of Neurobiology, Howard Hughes Medical Institute and Duke University Medical Center, Durham, NC 27710 USA; China National Genebank, BGI-Shenzhen, Shenzhen, 518083 China; Centre for GeoGenetics, Natural History Museum of Denmark, University of Copenhagen, Øster Voldgade 5-7, 1350 Copenhagen, Denmark; Trace and Environmental DNA Laboratory, Department of Environment and Agriculture, Curtin University, Perth, Western Australia 6102 Australia; Centre for Social Evolution, Department of Biology, University of Copenhagen, Universitetsparken 15, DK-2100 Copenhagen, Denmark

**Keywords:** Feather, Bird, Genome, Beta (β)-keratin, Alpha (α)-keratin, Evo-devo, Skin appendages

## Abstract

**Background:**

Vertebrate skin appendages are constructed of keratins produced by multigene families. Alpha (α) keratins are found in all vertebrates, while beta (β) keratins are found exclusively in reptiles and birds. We have studied the molecular evolution of these gene families in the genomes of 48 phylogenetically diverse birds and their expression in the scales and feathers of the chicken.

**Results:**

We found that the total number of α-keratins is lower in birds than mammals and non-avian reptiles, yet two α-keratin genes (KRT42 and KRT75) have expanded in birds. The β-keratins, however, demonstrate a dynamic evolution associated with avian lifestyle. The avian specific feather β-keratins comprise a large majority of the total number of β-keratins, but independently derived lineages of aquatic and predatory birds have smaller proportions of feather β-keratin genes and larger proportions of keratinocyte β-keratin genes. Additionally, birds of prey have a larger proportion of claw β-keratins. Analysis of α- and β-keratin expression during development of chicken scales and feathers demonstrates that while α-keratins are expressed in these tissues, the number and magnitude of expressed β-keratin genes far exceeds that of α-keratins.

**Conclusions:**

These results support the view that the number of α- and β-keratin genes expressed, the proportion of the β-keratin subfamily genes expressed and the diversification of the β-keratin genes have been important for the evolution of the feather and the adaptation of birds into multiple ecological niches.

**Electronic supplementary material:**

The online version of this article (doi:10.1186/s12862-014-0249-1) contains supplementary material, which is available to authorized users.

## Background

The integument of amniotes has evolved from a basic cornified epidermis for protection against the environment and the retention of water into an elaborate covering with epidermal structures used additionally for sexual display, camouflage, locomotion, and thermoregulation [1]. The claws, scales, beaks and feathers of reptiles and birds are formed from the products of two multigene families, alpha (α) and beta (β) keratins [2-6]. Alpha keratins, a subtype of intermediate filaments found in the epithelia of all vertebrates, have expanded and functionally diversified in amniotes through gene duplication [7]. The β-keratins are found exclusively in reptiles and birds and have also expanded and diversified especially in the avian and chelonian lineages [8-10].

The Type I (acidic) and Type II (basic/neutral) α-keratins form obligatory heterodimers [11,12] that make up the structural basis of the cornified epidermis and the epidermal appendages in mammals, such as wool, hair, claws, horns and hooves [6,7,13,14]. In birds, epidermal α-keratins make up the stratum corneum of the general epidermis and epidermal appendages such as the reticulate scale [15,16]. They are present in varying degrees along with the β-keratins in the avian scutate scales, claws, beaks, spurs, and lingual nails [6,14,17-21]. Although α-keratins are expressed in the early stages of feather development and in the cells of the rachis [22], the β-keratins make up 90% of the barbs and barbules of the mature feather [4,23-28]. In other words, the dynamic duplication and diversification of the β-keratin genes are thought to have contributed to the emergence of a novel epidermal appendage, the feather, which characterizes over 10,000 species of birds [8-10].

The avian β-keratins were originally grouped into four subfamilies (claw, feather, feather-like, and scale β-keratins) based on expression profiles and sequence heterogeneity [29-31]. More recently, an avian β-keratin isolated from cultured keratinocytes has been reported [32] and it is phylogenetically distinct from other β-keratin subfamilies [8,9,32-36]. This keratinocyte β-keratin is also found in crocodilians, but not in the squamates examined to date [9]. An additional β-keratin gene, BKJ, which is similar to feather-like β-keratins, has been identified on a unique locus and annotated as β-keratin from jun-transformed cells [8,9,37]. Thus, recent studies have regrouped the β-keratins into four different, but overlapping phylogenetically distinct subfamilies (claw, feather, scale, and keratinocyte β-keratins), proposing that the feather-like and BKJ genes are basal genes within the feather β-keratin clade [9].

The Type I and II α-keratins are found on two unlinked genomic loci. In mammals, the Type I α-keratin locus is separated by a small cluster of keratin associated proteins (KAPs). However this Type I mammalian locus still shows a high level of synteny with the green anole lizard, chicken and zebra finch which lacks the KAPs [7]. In birds, the Type I cluster is found on microchromosome 27 [7,38,39]. The Type II α-keratin cluster has been localized to linkage groups in the chicken and zebra finch genomes where they also show a high level of synteny with mammals and the green anole lizard [7]. One Type I gene variant is found on the Type II cluster suggesting a common genomic locus of origin for the α-keratins in amniotes [7].

All four β-keratin subfamilies (claw, feather, scale, and keratinocyte β-keratins) have been localized to a single locus in both the chicken and zebra finch; microchromosome 25. However, several other unlinked loci contain feather β-keratins [8]. Furthermore, the β-keratins from the green anole lizard are found on a single locus [10,36,40] and nearly half of the western painted turtle β-keratins are found on a single locus that is syntenic to microchromosome 25 of the chicken and zebra finch suggesting a common ancestral locus for β-keratins [10].

Here we have taken advantage of the sequencing of 48 bird genomes [41] spanning the avian phylogeny see companion study, [42] to investigate the evolutionary landscape of α- and β-keratins in the avian clade using copy number, molecular phylogenies, genomic orientation and transcriptome data. Our copy number data indicates that both α- and β-keratins have evolved in a dynamic manner with gene number contractions and expansions over the course of avian evolution leading to modern birds. Comparative transcriptome analyses demonstrate that 26 α-keratins and 102 β-keratins are differentially expressed in chicken scales and feathers during embryonic development. All four β-keratin subfamilies are highly expressed in developing scales, whereas the feather and keratinocyte β-keratins are highly expressed in the developing feather. The scales and feathers of birds have played important roles in the diversification of birds and their adaptation to multiple ecological niches. The dynamic evolution of the α- and β-keratins in the avian lineage accompanied these adaptations with the avian specific feather β-keratins making up to 85% of the total number of β-keratins, becoming the major structural component of the avian feather.

## Results

### Genome searches of α-keratins show lineage specific gene losses and gains

We searched the genomes of 48 avian species that span the avian phylogeny, 2 crocodilians and 2 turtles [41-44] for α-keratins and made use of the α-keratin copy number estimates for the green anole, human, opossum, house mouse and platypus from Vandebergh and Bossuyt [7] to test the hypothesis that there are no differences in copy number between birds and mammals and non-avian reptiles. We found that the total number of bird α-keratins, 26–38 ($\overline{x}$ = 31.271), is different from mammals and non-avian reptiles (fold change = 0.707) with 29–62 ($\overline{x}$ = 44.222) α-keratins (Additional file 1; [41]). Furthermore, birds have a lower number of Type I α-keratins (range 10–18; $\overline{x}$ = 14.292) than mammals and non-avian reptiles (range 13–35; $\overline{x}$ = 23.22; fold change = 0.616). The number of bird Type II α-keratins (range 11–22; $\overline{x}$ = 16.979) was also lower than that of mammals and non-avian reptiles (range 14–27; $\overline{x}$ = 21; fold change = 0.809).

The differences in copy number of Type I and II α-keratins among vertebrate groups suggest a dynamic gain or loss of this gene family. To test this hypothesis, we annotated all of the bird, crocodilian, turtle, human and green anole α-keratins to determine which α-keratin genes may have been lost in the avian lineage (Additional file 2). We applied the same nomenclature of α-keratins as the one based on human/mammalian genes [45]. We considered a gene to be lost in the avian lineage if it is present in human and at least one reptile and not found in any of the 48 bird genomes or avian expressed sequence tag (EST) libraries. Concurrently, we also identified the number of genes lost in the crocodilian and turtle lineages. Based on our annotations, we classified all avian and reptilian α-keratins into 17 Type I and 16 Type II genes (Table 1; tissue specificity in humans from references [45,46]). We found 8 Type I (see Table 1) and 6 Type II (see Table 1) α-keratins missing in birds. Interestingly, turtles have a relatively low number of Type I and II α-keratins and appear to have lost KRT10, KRT13, KRT31, KRT35 and KRT36 Type I genes and KRT1, KRT6A, KRT74, KRT79, KRT82 and KRT85 Type II genes. Crocodilians have also lost four of the same Type II genes, apparently independently (or possibly through incomplete lineage sorting of the common ancestor of turtles, birds and crocodiles) and lost the Type I gene KRT24. The cochleal or otokeratin Type II α-keratin cDNA was sequenced and described for the chicken by Heller *et al*. [47] and appears to have originated early in the reptilian lineage, as all reptiles and birds have at least one cochleal gene while none are found in humans.

**Table 1:** **Type I and II α-keratin expression in humans**

**Tissue**	**Type I α-keratins**	**Type II α-keratins**
Simple epithelium	KRT10*	KRT8
	KRT18	
	KRT19*	
	KRT20	
Stratified epithelium	KRT10*	KRT1
	**KRT13**	**KRT2**
	KRT14*	KRT5*
	KRT15*	KRT6A
	KRT16*	**KRT6B**
	KRT17*	
Corneal epithelium	KRT12	
Epithelium (nonspecific)	KRT23	KRT79
	**KRT24**	KRT80
	KRT42	
Hair	KRT14*	KRT5*
	KRT15*	**KRT72**
	KRT16*	KRT73
	KRT17*	**KRT74**
	KRT19*	KRT75
	**KRT28**	**KRT82**
	**KRT31**	KRT84
	**KRT35**	**KRT85**
	**KRT36**	

Table recreated from Schweizer *et al*. [45] and Moll *et al*. [46]. Gene names in bold text have been lost in birds.

*Indicates genes that are expressed in two of the tissues listed in the table.

While several α-keratin genes are absent in the avian lineage, at least one Type I and one Type II variant have expanded. KRT42, a Type I α-keratin found in epithelia of mammals has a mean copy number of 4.042 genes in birds and 1.143 genes in human and non-avian reptiles (fold change = 3.54). KRT75, a Type II α-keratin gene associated with a feather rachis anomaly [22], is higher in birds ($\overline{x}$ = 8.333) relative to humans or non-avian reptiles ($\overline{x}$ = 4.714; fold change = 1.76). At least two copies of KRT42 and KRT75 were found in all 48 birds.

The KRT75 expansion in birds led us to investigate whether an increase in copy number is related to mutations in KRT75 genes such as the mutation that causes the chicken frizzle feather phenotype [22]. The associated mutation is at the exon 5/intron 5 junction that induces a cryptic splice site found within exon 5 of KRT75 resulting in a 69 base pair deletion. This cryptic splice site has a similar nucleotide motif (GTGAAG) as that of the normal splice site at the exon5/intron5 junction. A total of 286 α-keratins were annotated as KRT75 genes for the 48 bird genomes, with most species having 5 or more variants (Additional file 2). Of these 286, we found that only 12 KRT75 genes have the GTGAAG motif from 7 species (wild turkey, chicken, medium ground finch, zebra finch, American crow, chimney swift and domestic pigeon). Unexpectedly, these 7 species all had below the mean KRT75 copy number (8.333). These data suggest that the frizzle feather phenotype should be rare among birds. Indeed, only chickens, pigeons, geese and canaries have been described as having feather characteristics similar to the frizzle phenotype [48]. This is consistent with our finding that the pigeon has the cryptic splice site as well as the two finches, which are in the same Family as canaries.

### Avian adaptations to novel lifestyles was accompanied by β-keratin gene family dynamics

From all 48 bird genomes combined, we found 1623 complete β-keratin genes with both start and stop codons and without unknown sequence (−NNN-) or frame shift mutations. We also found 1084 incomplete avian β-keratins (Additional file 2). This analysis showed extreme variation in copy number for birds with the barn owl having only 6 β-keratins and the zebra finch having a maximum of 149 complete genes (Additional file 1; [41]). Consistent with earlier studies [9,10], the American alligator and green sea turtle have 20 and 26 β-keratins, respectively. While, the barn owl and zebra finch represent the minimum and maximum number of total β-keratins, respectively, we found that the mean number of β-keratins in birds was 33.81. We identified 4 statistical outliers (zebra finch: 149, chicken: 133, pigeon: 81 and budgerigar: 71) that have a value greater than or equal to the third quartile plus 1.5 times the IQR (interquartile range), which are birds with the highest number of β-keratin genes (Additional file 1; [41]). These drastic copy number differences may relate to the quality of the genome build or to other factors such as domestication, since they belong to 4 of 5 domesticated species among the 48 birds.

Annotation of the β-keratins was performed using the Greenwold and Sawyer [8] dataset. The feather-like β-keratins and β-keratins from jun-transformed cells (BKJ) have been shown to group with feather β-keratins in previous phylogenetic analyses [9], therefore genes resulting in a best hit to those genes were annotated as feather β-keratins. We found that feather β-keratins comprised up to 85% of the total number of β-keratins for birds. Using Levene’s test we can reject the null hypothesis of equal variance among claw (W = 23.442, p-value < 0.001), scale (W = 23.107, p-value < 0.001), keratinocyte (W = 21.744, p-value < 0.001) and total number of β-keratins, but not for feather β-keratins and the total number of β-keratins (W = 0.479, p-value = 0.491), indicating that the variance of feather β-keratin copy number for birds can be used as an indicator of the variance in the total β-keratin copy number for these 48 bird species.

In order to ascertain if β-keratin copy number differences in birds correlate to species phylogenetic relatedness or lifestyle (aquatic and semi-aquatic and predatory; Additional file 1; [41], Additional file 3; [42]), we calculated the proportion of each of the four β-keratin subfamilies (Additional file 2) to the total number of β-keratins for each lifestyle using standard and phylogenetic ANOVA. We found that the proportion of feather β-keratins to the total number of β-keratins is significantly lower for aquatic and semi-aquatic birds (see Additional file 1; [41]) than for land birds (F_1,46_ = 7.84; standard ANOVA p = 0.007; phylogenetic ANOVA p = 0.029), while the proportion of keratinocyte β-keratins is significantly higher for aquatic and semi-aquatic birds than land birds (F_1,46_ = 10.79; standard ANOVA p = 0.002; phylogenetic ANOVA p = 0.013). This includes aquatic and semi-aquatic species that are considered to have been independently derived according to the genome scale phylogeny in our companion study (Jarvis *et al.* [42]; Additional file 3). However, the species phylogeny (Additional file 3; [42]) indicates that the eagles do not group with the other aquatic birds, we therefore removed them from the aquatic bird list and found that only the higher proportion of keratinocyte β-keratins remained statistically significant (F_1,46_ = 6.84; standard ANOVA p = 0.012; phylogenetic ANOVA p = 0.043) indicating more strongly that the change in β-keratin gene numbers could be associated with an aquatic lifestyle. We next considered birds with a predatory lifestyle (see Additional file 1; [41]) and found that the proportion of claw β-keratins (F_1,46_ = 6.75; standard ANOVA p = 0.0126; phylogenetic ANOVA p = 0.033) and keratinocyte β-keratins (F_1,46_ = 5.77; standard ANOVA p = 0.02; phylogenetic ANOVA p = 0.047) is significantly higher in predatory birds than other birds while the proportion of feather β-keratins (F_1,46_ = 7.81; standard ANOVA p = 0.008; phylogenetic ANOVA p = 0.022) is significantly lower for predatory birds. Like the aquatic species, this finding occurs for independent lineages of predatory birds. We did not find any significant differences in copy number for the four β-keratin subfamilies between the major stem lineages of birds: Paleognathae vs. Neognathae or Paleognathae and Galloanserae vs. Neoaves; see also Jarvis *et al.* [42]). Together these data indicate that dynamic changes in the proportion of β-keratin subfamilies have occurred as birds have adapted to novel lifestyles.

### The α- and β-keratin multigene families have similar patterns of sequence divergence following gene duplication

**Figure 1:**
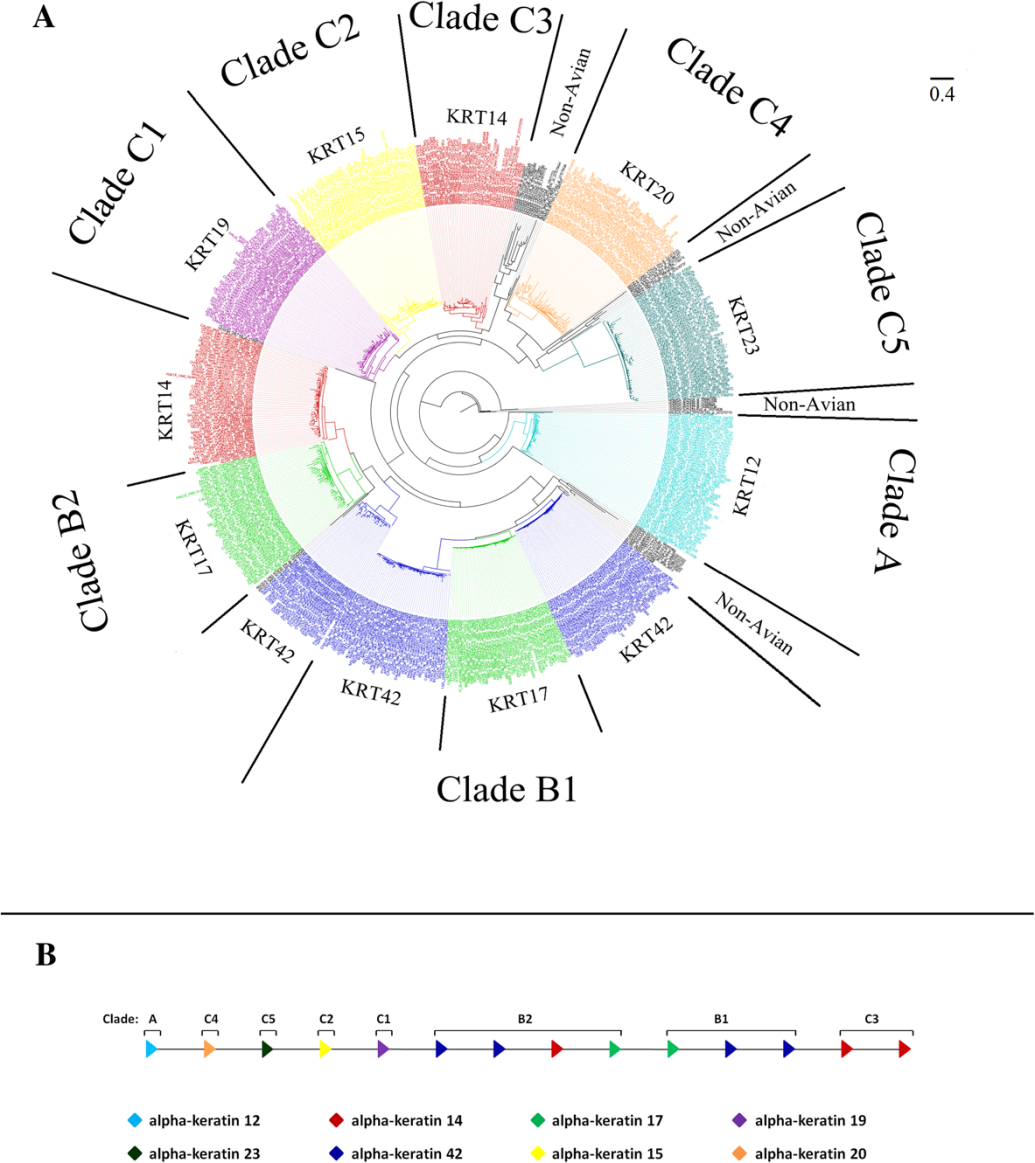
**Molecular phylogeny and proposed genomic orientation of Type I α-keratins.** Part **A** is the maximum likelihood phylogeny of Type I α-keratins from human, green anole lizard, green sea turtle, American alligator and the 48 birds. Annotation of Type I α-keratins is based upon avian gene annotations. All clades are statistically significant. Genes labeled as non-avian include genes from human, green anole lizard, green sea turtle and/or American alligator. Part **B** is the proposed genomic orientation of Type I α-keratins in birds. While this whole region was not found on a single continuous genomic scaffold for some birds, the genomic alignment of scaffolds/contigs with at least 2 different gene variants resulted in this proposed consensus gene orientation of birds. Annotations are based on Part **A**. The direction of the arrow is indicative of the DNA strand.

**Figure 2:**
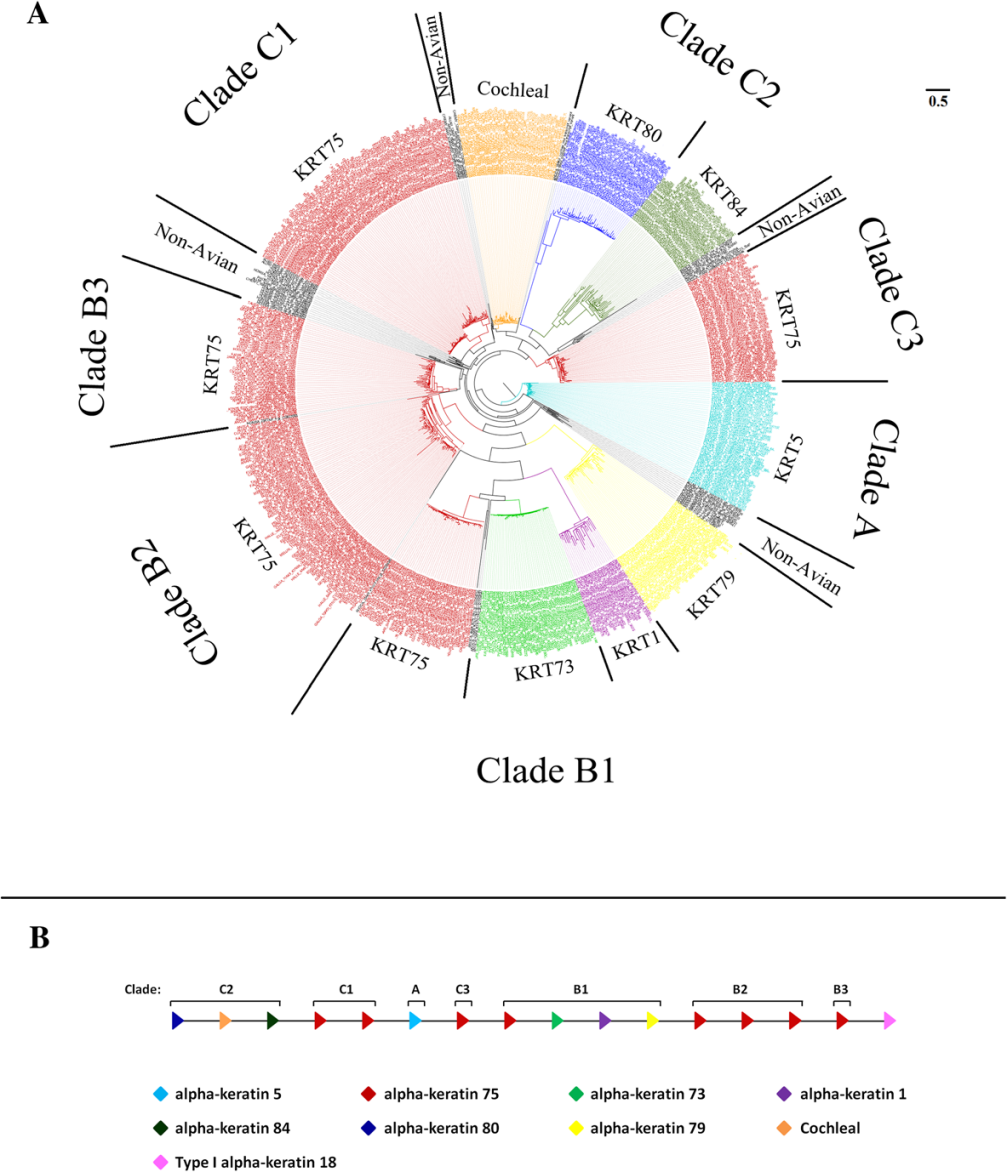
**Molecular phylogeny and proposed genomic orientation of Type II α-keratins.** Part **A** is the maximum likelihood phylogeny of Type II α-keratins from human, green anole lizard, green sea turtle, American alligator and the 48 birds. Annotation of Type II α-keratins is based upon avian gene annotations. All clades are statistically significant. Genes labeled as non-avian include genes from human, green anole lizard, green sea turtle and/or American alligator. Part **B** is the proposed genomic orientation of Type II α-keratins in birds. While this whole region was not found on a single continuous genomic scaffold for some birds, the genomic alignment of scaffolds/contigs with at least 2 different gene variants resulted in this proposed consensus gene orientation of birds. Annotations are based upon Part **A**. The direction of the arrow is indicative of the DNA strand. The one Type I α-keratin, KRT18, shown in the consensus genomic orientation was only found in 7 species of birds, but was included in this figure based on the present data and previous studies [7,38,39].

**Figure 3:**
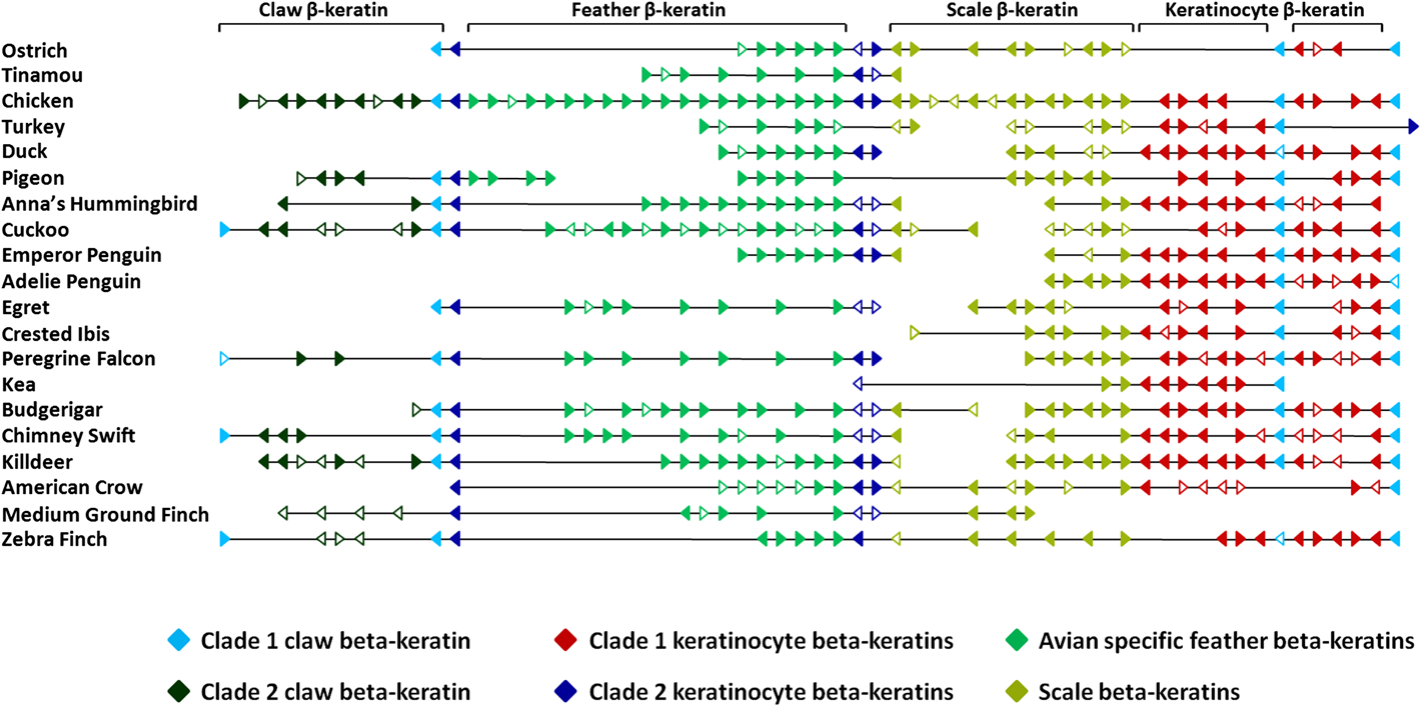
**Genomic orientation of β-keratins in birds.** This figure is a genomic alignment of β-keratins in birds containing a genomic locus with at least two β-keratin subfamilies. For the chicken and zebra finch this locus is microchromosome 25. Although feather β-keratins can be found on many genomic loci other than the one shown here [8], we focused on this locus as it has members from all of the β-keratin clades. The annotations are based on our β-keratin phylogeny (Additional file 4; [41]). The breaks in the line for each species are indicative of different genomic scaffolds. The direction of the arrow is indicative of the DNA strand. The arrows with solid colors are complete genes and those with white centers are incomplete genes. The annotation above the figure is based on each of the four β-keratin subfamilies, while the clades based on the β-keratin phylogeny (Additional file 4; [41]) are shown below the figure.

To further our understanding of the evolution of the α- and β-keratin multigene families in birds we performed phylogenetic analyses and examined their genomic orientation (Figures 1, 2 and 3, Additional file 4; [41]). By examining these two types of data we were able to elucidate the gene duplication history of multigene families and gain a deeper understanding of their genomic origin.

The phylogenetic analyses of the Type I and II α-keratins (Figures 1A and 2A) demonstrate that they can be separated into 3 main clades (Clade A, B and C) with Clade A being composed of a single basal gene. The remaining two clades (Clade B and C) of the Type I and Type II α-keratins are composed of multiple phylogenetically significant sub-clades of different gene variants. The α-keratins genes, KRT75 and 42 are distributed among several sub-clades in their respective phylogenies (Figures 1A and 2A) and the genes in different sub-clades are generally found interposed between other α-keratin genes on their respective α-keratin loci (Figures 1B and 2B). These phylogenies clearly demonstrate that KRT42 and KRT75 genes have expanded and their duplication history is marked by non-tandem duplication and subsequent sequence divergence. Our phylogenetic and genomic orientation data further support the idea that Type I and II α-keratins have evolved through gene duplications in a concerted fashion [49].

For the β-keratin phylogeny, the green anole forms a clade and therefore was selected as the outgroup (Additional file 4; [41]). Furthermore, a clade of keratinocyte β-keratins composed of bird, turtle and crocodilian genes forms the basal sister clade (Clade 1 keratinocyte β-keratins, Additional file 4; [41]). The remaining bird, turtle and crocodilian β-keratins form a second major clade that is composed of another keratinocyte β-keratin clade (Clade 2 Keratinocyte β-keratins), two claw β-keratin clades, one scale β-keratin clade and an avian specific feather β-keratin clade. While all of the scale β-keratins annotated in Additional file 4 [41] group together, only a portion of them form a phylogenetically significant clade with strong bootstrap support. Similar to the α-keratin genes, KRT42 and KRT75, the β-keratin subfamilies, keratinocyte and claw β-keratins, have duplicated in a non-tandem pattern (Figure 3) and form multiple phylogenetically significant clades (Additional file 4; [41]). Collectively, these results indicate that the transposition of duplicated genes on the same locus in a non-tandem fashion is adequate to induce a relatively high level of sequence divergence possibly resulting in neofunctionalization.

Generally our gene trees (Figures 1A, 2A and Additional file 4; [41]) did not follow the genome scale phylogeny of the 48 bird species in Jarvis *et al.* [42] (Additional file 3). This is not surprising because in that study no single gene tree they analyzed was identical to the species tree. Due to incomplete lineage sorting, most genes differed from the species tree by 20% of the branches, and our findings above indicate large scale convergence of keratins among aquatic and predatory birds with relationships different from the species tree. For each gene phylogeny (Figure 1A, 2A and Additional file 4; [41]), however, we found that sub-clades of closely related species frequently grouped together such as those of the Palaeognathae (tinamou and ostrich), Galloanseres (chicken, turkey and duck) and some species of the Psittacopasserimorphae (songbirds and parrots).

The phylogenetic analyses of Type I and II α-keratins (Figures 1A and 2A) support the interpretation that birds have lost 14 α-keratin gene variants (see Table 1). We found that the gene variants that are missing in birds form statistically significant clades in human and non-avian reptiles. Additionally we found that our phylogenetic analyses resulted in slightly different copy number counts for the α-keratin genes and β-keratin subfamilies from the annotations detailed above (see Additional file 2). However, the statistical significance of the comparisons between the β-keratin subfamilies and lifestyles largely remained valid with the phylogeny data (data not shown). The exception is with the keratinocyte β-keratin comparison between aquatic and land birds (F_1,46_ = 5.76; standard ANOVA p = 0.021; phylogenetic ANOVA p = 0.057) when the eagles are grouped with land birds.

Previous studies [7,38,39] have shown that one Type I α-keratin gene (KRT18) is found on the Type II α-keratin locus for fish, amphibians, mammals and the green anole lizard indicating that the α-keratins evolved from a single locus. While Vandebergh and Bossuyt [7] found a Type I α-keratin on chromosome unknown of the chicken, they found no direct evidence that Type I and II α-keratins are linked in birds. In fact the gene they found on chicken chromosome unknown was not found during our genome searches. However, we did find that 8 of the bird species (chimney swift, common cuckoo, little egret, peregrine falcon, crested ibis, brown mesite, white-throated tinamou and common ostrich), the 2 crocodilian species and the western painted turtle had one Type I α-keratin on a Type II locus (Figure 2B). Furthermore, these genes were annotated as KRT18. The green sea turtle and green anole lizard both have 2 KRT18 genes, which are found on 2 different loci.

### Differential expression of the α- and β-keratin genes in chicken epidermal tissue during embryogenesis

We performed transcriptome analyses of scale, dorsal feather and wing feather tissues during chicken development using a customized version of the chicken 44 K Agilent microarray [50]. We customized the microarray chip by adding all 27 α-keratins and 102 of the 133 chicken β-keratins. The number of unique 60-mer oligonucleotides of β-keratins was constrained due to the highly repetitive nature of feather β-keratins and thus we were only able to produce unique oligonucleotides for 68 of the 99 chicken feather β-keratins.

Tissue samples from the chicken scutate scale, dorsal feather and wing feather were from embryonic day 17 and 19 and scutate scale and dorsal feather at day 8. Although feather morphogenesis begins as early as day 6.5 to 7, the cellular differentiation of barbs and barbules and the accumulation of β-keratin does not begin until ~ day 12 of embryogenesis [2,4,51,52]. Scutate scale morphogenesis does not begin until day 9.5 of embryogenesis, and β-keratin accumulation is not detected until 15–16 days of development [16,53-56]. Thus, we selected day 8 for the initial sampling of the scale and feather tissues [53].

Comparison of day 8 scutate scale and day 8 dorsal feather tissues showed that 6 α-keratins and 18 β-keratins were differentially expressed, but the fold change values were 5 or below (Table 2 and Additional file 5). Comparisons of day 8 and day 17 had the largest number of differentially expressed α- and β-keratins. In comparing day 8 dorsal feather to day 17 dorsal feather we found 20 up-regulated and 2 down-regulated α-keratins in the day 17 dorsal feather. Also 98 β-keratins were up-regulated in day 17 dorsal feather, which showed up to an 112,000 fold change for a keratinocyte β-keratin and a 5,000 fold change for a feather β-keratin. We found 23 α-keratins that were up-regulated and 2 α-keratins that were down-regulated in day 17 scutate scale while 101 β-keratins were up-regulated in day 17 scale (Table 2). Day 8 and 19 comparisons of the dorsal feather and scutate scale had slightly lower numbers of α- and β-keratin indicating that day 17 of development may be the peak level of keratin expression.

**Table 2:** **Expression of α- and β-keratins during embryonic chicken development**

**Sample comparisons (Tissue/embryonic day of development)**	**Number of differentially expressed α- and β-keratins**					
	Type I α-keratins*	Type II α-keratins*	Keratinocyte β-keratins*	Scale β-keratins*	Claw β-keratins*	Feather β-keratins*
DF8/DF17	11[↓]	2[↑]; 7[↓]	10[↓]	9[↓]	12[↓]	67[↓]
DF8/DF19	11[↓]	1[↑]; 8[↓]	9[↓]	5[↓]	8[↓]	61[↓]
DF17/DF19	2[↑]	0	3[↑]	8[↑]	10[↑]	67[↑]
DF17/WF17	5[↓]	1[↓]	2[↓]	7[↓]	0	3[↓]
DF19/WF19	1[↑]; 1[↓]	3[↑]; 1[↓]	0	8[↓]	8[↓]	5[↓]
SC8/DF8	1[↑]; 1[↓]	3[↑]; 1[↓]	0	2[↑]	0	13[↑]; 1[↓]
SC8/SC17	1[↑]; 12[↓]	1[↑]; 10[↓]	10[↓]	9[↓]	12[↓]	70[↓]
SC8/SC19	1[↑]; 13[↓]	1[↑]; 8[↓]	10[↓]	9[↓]	12[↓]	65[↓]
SC17/DF17	4[↑]; 2[↓]	6[↑]	6[↑]; 1[↓]	9[↑]	11[↑]	17[↑]; 8[↓]
SC17/SC19	1[↑]; 1[↓]	2[↑]	4[↑]	3[↑]	1[↑]	35[↑]
SC17/WF17	4[↑]; 1[↓]	6[↑]; 1[↓]	9[↑]; 1[↓]	4[↑]; 4[↓]	12[↑]	17[↑]; 21[↓]
SC19/DF19	7[↑]; 1[↓]	5[↑]	7[↑]; 2[↓]	9[↑]	11[↑]	26[↑]
SC19/WF19	6[↑]; 1[↓]	4[↑]	4[↑]; 3[↓]	4[↑]	10[↑]	3[↑]; 1[↓]
WF17/WF19	1[↑]	0	1[↑]	0	0	5[↑]

Number of differentially expressed α- and β-keratins during embryonic chicken development for 14 sample comparisons with a p-value cutoff of 0.05 and a fold change cutoff of 2.0. DF, dorsal feather; WF, wing feather; SC, scale.

*Direction of selection is indicated in brackets (↑: Up-regulated; ↓: Down-regulated) after each copy number and refers to the first sample for each comparison.

Although the scale β-keratins were annotated based upon their expression in scale tissue [30], it appears that the claw β-keratins are expressed at the highest level in scale tissue. In the scutate scale comparisons of day 8 vs. 17 and day 8 vs. 19, 7 out of the 10 highest fold changes (up-regulated genes) in day 17 and 19 scutate scale are claw β-keratins. Additionally, the day 17 and 19 scutate scale inter-tissue comparisons (dorsal and wing feather) showed that 9 of the highest fold changes (up-regulated genes) in the scutate scale are claw β-keratins indicating that claw β-keratins have an important role in the composition of epidermal appendages, such as scales, in addition to the claw and beak [8].

Four sample comparisons had genes from the feather β-keratin subfamily that were up and down regulated (Table 2). The day 8 comparison of the scutate scale and dorsal feather indicates that a single feather β-keratin from microchromosome 27 is up-regulated in the day 8 dorsal feather while all of the down-regulated feather β-keratins are on different loci (chromosome 1, 2, microchromosome 25, and chromosome unknown). Furthermore, feather β-keratins on microchromosome 27 are up-regulated in day 17 scutate scale in comparisons of the scutate scale day 17 and dorsal and wing feather day 17. Additionally, comparisons between the scutate scale and wing feather during day 19 of embryogenesis show an up-regulation of microchromosome 27 feather β-keratins and down-regulation of feather β-keratins on other loci in the day 19 scutate scale tissue. These data indicate that feather β-keratins on microchromosome 27 are regulated differently from feather β-keratins on other loci (chromosome 1, 2, 6, 10, and unknown and microchromosome 25).

We found that the basal BKJ genes of the feather β-keratin clade (Additional file 4; [41]) are expressed at a higher level in the dorsal and wing feather when compared to the scutate scale at day 17 and 19 (Additional file 5). Although BKJ genes are expressed in higher levels in the feather, they are also expressed in the scutate scales as evidenced by the down-regulation in the scutate scale comparisons between day 8 and day 17 and 19. The feather-like β-keratins are found in multiple comparisons indicating they are expressed in both feather and scutate scale tissue. Interestingly, the only three feather β-keratins expressed in dorsal and wing feather comparisons at day 17 are the feather-like β-keratins suggesting that they have an important role in feather morphology. While the feather-like β-keratins are linked to other feather β-keratin genes on chromosome 25, the BKJ genes are found on chromosome 6 and are not linked to any other β-keratins indicating that intra and inter-locus differential expression occurs among the feather β-keratin clade.

**Figure 4:**
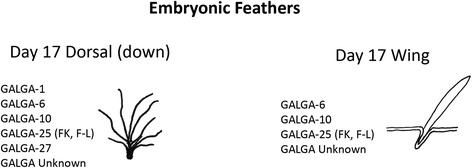
**Expression of feather β-keratins during embryonic feather development.** This figure summarizes the present data on the expression of feather β-keratin by chromosomal location in embryonic (day 17 down and wing feathers) feathers using data from the present study. For each feather type (Day 17 down, Day 17 Wing), the chicken (GALGA) chromosome number of the feather β-keratins expressed is listed. The feathers of day 17 dorsal skin express feather β-keratins located on GALGA chromosome 1, 6, 10, 25 (both feather and feather-like β-keratins) and chromosome unknown. Wing feathers, at day 17, express feather β-keratins from GALGA chromosome 6, 10, 25 (both feather and feather-like β-keratins) and chromosome unknown.

Feather β-keratins in the chicken genome are found on multiple loci (Additional file 4; [41]) [8]. Based on our sample comparisons in this study we were able to determine which feather β-keratins from which chicken genomic loci (GALGA, chromosomes) were being expressed in the dorsal and wing feathers during embryonic development. The genomic loci of feather β-keratins being expressed in day 17 dorsal (down) feathers and day 17 wing feathers are summarized in Figure 4. The feather β-keratins expressed in the day 17 down feathers are located on GALGA 1, 6, 10, 25 and 27. In addition, the feather-like genes on GALGA 25 were expressed as was feather β-keratin on GALGA unknown. The feather β-keratins expressed in the day 17 wing feathers are located on GALGA 6, 10, and 25. In addition, the feather-like genes on GALGA 25 were expressed as was feather β-keratin on GALGA unknown (Figure 4).

The only comparison showing both up and down-regulated scale β-keratins is the day 17 scutate scale versus wing feather, which indicates that scale genes annotated as 1, 2, 3 and 5 are up-regulated while scale 7, 8, 9 and 10 are down-regulated. These scale β-keratins are all found on the same locus (GALGA 25) and their number describes their orientation in a 5′ to 3′ direction. Alternatively, only one keratinocyte β-keratin (GALGA25_Ktn6) is consistently differentially expressed from the other keratinocyte β-keratins on GALGA 25. These results indicate that while β-keratins from all subfamilies are being expressed in these tissues, intra-locus differential expression of GALGA 25 β-keratins and inter-locus differential expression of feather β-keratins may contribute to the structural complexity of these and other avian epidermal appendages.

## Discussion

This study made use of the newly published genomes of 45 birds in addition to the 3 previously published bird genomes (chicken, zebra finch and turkey) to investigate the multigene families of α- and β-keratins [41]. Incomplete or low coverage genomes can lead to an underestimate of gene family copy numbers. For α-keratins, little variation is seen among our copy number estimates (Additional file 1; [41]). Furthermore, based on the α-keratin annotation, we find consistent copy number estimates of the different Type I and II α-keratins (Additional file 2). In contrast to the α-keratins, the β-keratins have a much larger variation in copy number estimates (Additional file 1; [41]). While both of these gene families are tandemly arrayed on at least 2 genomic loci strong differences exist in the variation of the copy number estimates among the 48 birds. The newly sequenced bird genomes are separated into two coverage groups; low (<50× coverage) and high (>50× coverage) [41]. The coverage in these two groups vary, but if the β-keratin copy number is related to genome coverage the copy number of β-keratins should correlate with fold coverage and contig and scaffold N50. However, for each fold coverage group (low and high) we do not find a statistically significant correlation between β-keratin copy number and fold coverage, contig N50 or scaffold N50. While, we do not discount the likelihood that some of the bird species in this study have unsequenced β-keratins, we believe that the relative variation in β-keratin copy number among birds is appropriately represented in this study.

### Alpha (α)-keratins

The α-keratin nomenclature used in this study is based upon mammals and more specifically humans [45]. While mammals have shown the largest expansion of α-keratins among amniotes [7], we find that there is avian specific gene loss and gain of α-keratins. The expansion of specific α-keratin gene variants (KRT42 and KRT75) in birds may not be the result of gene duplication of a single “parent” gene, but instead the duplication of several different gene variants resulting in novel α-keratins of avian origin. If some of these genes are novel α-keratins as the phylogeny and genomic alignment data indicate, then the current α-keratin nomenclature, based on mammalian genes, does not adequately account for the diversity found in birds. Therefore, we suggest that the KRT42 genes be annotated as KRT42a and b and the KRT75 genes be annotated as KRT75a-e to reflect their phylogenetic relationship and genomic orientation.

Our discovery of the KRT42 and KRT75 expansion in the avian lineage and Ng *et al.* [22] discovery that KRT75 is important in feather rachis development indicates that the duplication of KRT42 and KRT75 α-keratins may be the result of concerted evolution and that together they form the α-keratin heterodimer in feathers. Furthermore, it is likely that these duplicated genes contributed to the evolution of feathers as did the feather β-keratins [57].

### Beta (β)-Keratins

The fact that the extreme statistical outliers from the average number of ~34 β-keratins across bird species are all species that have undergone various degrees of domestication (zebra finch, chicken, pigeon and budgerigar), indicate that there could be an association between these observations. In support of this relationship, both the Peking duck (46) and turkey (46), the remaining domesticated species among the birds, have an above average number of β-keratins. Given that domestication may increase recombination rate [58,59], the extreme variation in β-keratin copy numbers among birds may be partially linked to higher recombination rates on β-keratin loci and the domestication of these species. The differential expression of feather β-keratins is related to their genomic locus [57], signifying that expansion of feather β-keratins, through unequal crossing over events on specific loci, may be induced by artificial selection.

### Expression of feather β-keratins and the evolution of feathers

Numerous studies have examined the biochemical and molecular make up of embryonic and adult feathers, as well as their component parts [16,23,24,26-28,32,34,35,56,57,60-64]. For example Kemp [62] suggested that there were 25–35 different feather keratin mRNA molecules in the embryonic feather and a total of 100–240 keratin genes in the chicken genome. The present study supports the view that a high number of α- and β-keratins are expressed during the embryonic development of scutate scales and feathers in the chicken (Table 2). These results are further supported by a recent study by Ng et al. [57], which found that 90% of α-keratin and over 95% of β-keratin genes in the chicken are differentially expressed during post-hatching feather genesis. However, the number of β-keratins that can be extracted from the cornified tissues of scales and feathers and detected on 2-dimensional gels is considerably smaller [14,21,55,63-65], suggesting that messenger RNAs are being inactivated, perhaps by microRNAs [66].

Recently, Kowata *et al.* [28] found that the feather β-keratin on chromosome 7 of the chicken (GALGA 7) is expressed in the cells that form the barbules of pennaceous feathers but is not expressed in the barbules of plumulaceous feathers. In the present study, we did not find any differential expression of the GALGA 7 feather β-keratin in embryonic feathers supporting the results of Kowata *et al.* [28]. However, we did find that GALGA 27 feather β-keratins are differentially expressed in comparison to feather β-keratins on other loci and that GALGA 27 feather β-keratins are generally up-regulated in scale tissue. Previous studies have demonstrated that the ancestral locus of β-keratins is homologous to GALGA 25 of the chicken [8-10], suggesting that feather β-keratins diversified to other genomic loci through duplication and translocation. Recently, Ng et al. [57] examined which genomic loci (chromosome) are utilized for the expression of feather β-keratins in post-hatched contour and flight feathers. While it is clear that feather β-keratin located on GALGA 7 is only expressed in the barbules and possibly the hooklets of pennaceous feathers, feather β-keratins from multiple loci are expressed in the ramus, rachis, and calamus of post-hatched feathers. Overall these data suggest that as the avian epidermis evolved to produce novel structures (such as pennaceous feathers) it took advantage of the diversity of feather β-keratins that evolved on different loci.

### Evolution of birds into novel ecological niches

Bird diversification is marked by evolution into novel habitats and ways of life such as predatory and aquatic lifestyles. Birds of prey are identified by their powerful beaks and claws. In the case of the claw, studies indicate that the morphology of the claw of birds of prey (also referred to as a talon) differs from non-raptorial birds and between different Orders of birds of prey [67,68] (however see Birn-Jeffery *et al.* [69]). In addition to being expressed in the claw [31], claw β-keratins are also expressed in the beak of the chicken [8,70]. In this study we found that the birds of prey have a significantly higher proportion of claw β-keratins than non-raptorial birds, which may indicate that they have played an important role in the evolution of these unique epidermal appendages.

The feathers of aquatic birds have been shown to have a higher hydrophobicity than the feathers of terrestrial birds [71]. This may be important for thermal regulation especially for birds in adverse climates, such as penguins. Our analysis of β-keratin copy number variation among birds has shown that the proportion of keratinocyte β-keratins is higher and the proportion of feather β-keratins is lower for aquatic birds compared to terrestrial birds. Also, we found that at least 98 of the 133 chicken β-keratin genes are transcribed during the formation of feathers in the chicken. While feather β-keratins are annotated based upon the tissue in which their amino acid sequence was first determined [72], it has been shown that there are actually multiple β-keratin gene variants (subfamilies) expressed during embryonic development of feathers [9,29,30]. Collectively, this indicates that the proportion of gene variants is important as birds have adapted to their lifestyles (aquatic, terrestrial, predatory) and that their feather, claw and beak structure may have been modified by the dynamic expansion and contraction of specific β-keratin gene variants.

## Conclusion

**Figure 5:**
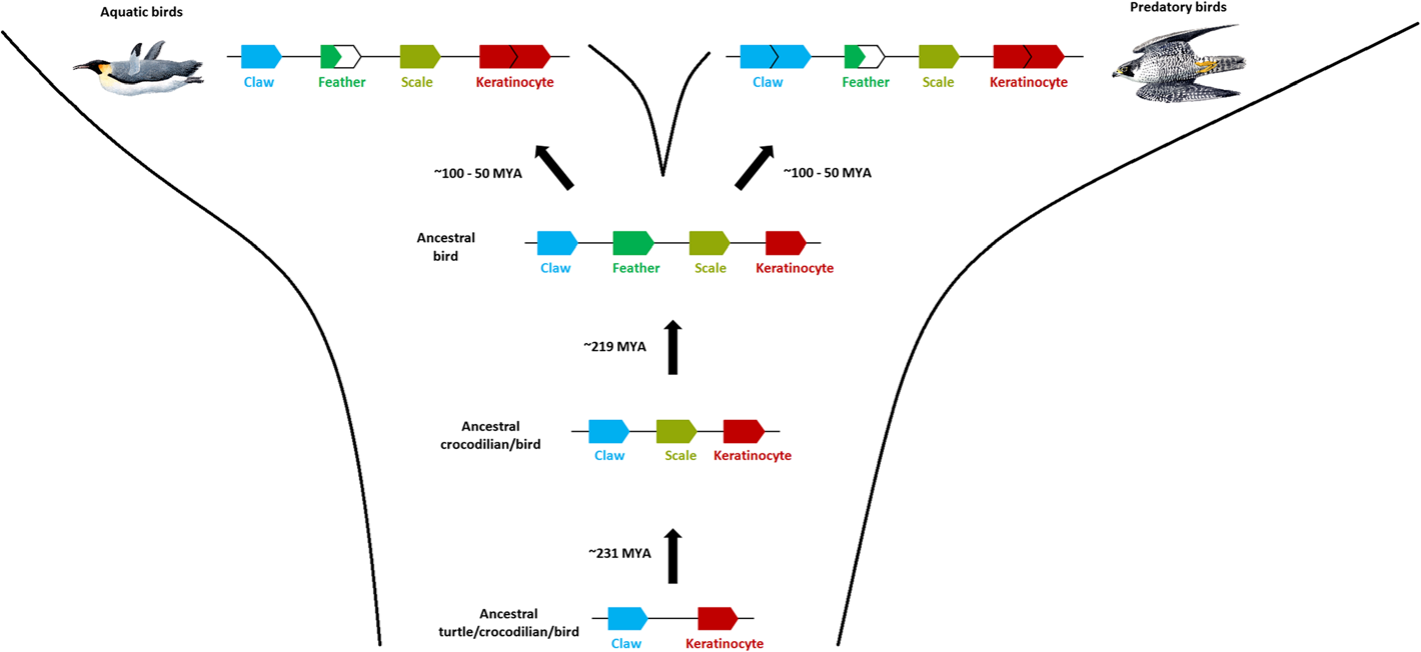
**Dynamic evolution of β-keratins in the archosaur lineage.** This figure illustrates a proposed scenario of β-keratin evolution on the ancestral locus in archosaurs. The bottom row is the proposed locus of β-keratins in the ancestor of turtles, crocodilians and birds. The second row from the bottom indicates that the scale β-keratins have emerged since the divergence of turtles from crocodilians and birds. The origin of the feather β-keratins occurs after the divergence of crocodilians and birds. The order of the β-keratins subfamilies is based on our genomic data from the 48 birds and green sea turtle. The top row illustrates the dynamic changes of the proportions of β-keratin subfamilies in modern birds with aquatic and predatory lifestyles. Both aquatic and predatory birds have a larger proportion of keratinocyte β-keratins and smaller proportion of feather β-keratins in their genomes. Additionally, predatory birds have a larger proportion of claw β-keratins. The divergence times are in millions of years ago (MYA). The divergence estimates of the turtle – crocodilian/bird split and the crocodilian – bird split are from Shedlock and Edwards [73]. The divergence time estimates for birds is from Jarvis et al. [42] and is the range starting with the divergence of the Palaeognathae and Neognathae (~100 MYA) and the subsequent divergences of most ordinal groups by ~50 MYA. Jon Fjeldså produced the images of the birds (emperor penguin on the left and peregrine falcon on the right).

The number of α-keratin genes is reduced in the avian lineage, and while still important for feather development, for example during rachis morphogenesis [22], their low abundance in the barbs and barbules of feathers demonstrates that they have a reduced role in establishing the composition of mature feathers. On the other hand, the β-keratin multigene family has undergone dramatic expansions in the bird and turtle lineages resulting in novel epidermal appendages [8-10]. Members of all β-keratin subfamilies are expressed during the development of scutate scales and feathers with feather β-keratins becoming specialized in their expression profiles in the diverse assortment of feathers found in present day birds [28,57] (Figure 4). The early evolution of β-keratins in the archosaurian lineage is marked by lineage specific expansions, but differences in the proportion of claw, feather, and keratinocyte β-keratin genes in modern birds may be attributed to their ecological niche (Figure 5). Our overall findings suggest that the number of β-keratins and the relative proportion of β-keratins in each subfamily influenced the composition of avian skin appendages and therefore their structural properties. Clearly, the evolution of feathers in the lineage leading to modern birds has been shaped by the dynamic evolution of α- and β-keratins.

## Methods

### Genome searches

The genome build information and statistics for the birds used in this study are detailed in Zhang *et al*. [41]. Additional file 1 is reprinted with permission from Zhang *et al*. [41] and lists the English names for all species used in this study, while scientific Latin species names are listed in Additional file 2.

Alpha keratin sequences for the chicken, green anole and human were downloaded via NCBI and their accession numbers and names are listed in Additional file 6. The copy number and position of these sequences coincide with the results reported in Vandebergh and Bossuyt [7]. Genome searches were conducted using standalone BLAT (v35) fast sequence search command line tool [74] and GeneWise (v2.2.0) was used as a homology based predictor of gene structure [75] with chicken and green anole α-keratin sequences downloaded via NCBI (Additional file 6). Type I and II α-keratin searches were conducted separately. BLAT hits with a Match score, the number of matches minus the mismatches, of ≥ 250 were used to select the best hits for all α-keratin genes. Selected regions were extracted and gene model structures were predicted using GeneWise (v2.2.0). Bird genomes were searched using chicken α-keratins and the alligator and green sea turtle genomes were searched using both the chicken and green anole α-keratins.

Beta keratin searches of the bird genomes were performed using 18 peer reviewed avian β-keratins sequences from the Swiss-prot database (Additional file 6). These sequences were used to perform a TBLASTN (v2.2.19) search and GeneWise (v2.2.0) was used as a homology based predictor of gene structure [75]. Avian β-keratins were aligned to the bird genomes using TBLASTN. Aligned regions were selected using the most similar query β-keratin with a length not less than 50% of the protein query. Selected regions were extracted and gene model structures were predicted using GeneWise (v2.2.0). For the green sea turtle genome, we performed a TBLASTN (v2.2.19) search using the Greenwold and Sawyer [8] data set, which included the crocodilian β-keratins. Following gene selection, we refined and filtered the genes to exclude sequences that did not have an open reading frame (ORF), contained unknown sequence (−NNN-) within the ORF, were less than 80 amino acids, or contained frameshift mutations. The American alligator and green anole β-keratin genes were obtained from Greenwold and Sawyer [9] and Dalla Valle *et al*. [35], respectively.

### Gene annotation

Type I and II α-keratin gene annotations were performed using the standalone BLAST+ program and the non-redundant GenBank CDS translations+PDB+SwissProt+PIR+PRF database downloaded February 2013. The β-keratin annotations were performed using the standalone BLAST+ program and the Greenwold and Sawyer [9] dataset as a database.

Annotation of the avian α-keratins from the 48 bird genomes indicated that several Type I and II α-keratins are not found in birds. In order to verify these findings we downloaded all available EST libraries for the birds used in this study and used the standalone BLAST+ program and tblastn to search the expressed sequence tag (EST) libraries of the chicken (603,076 ESTs), turkey (17,435 ESTs), duck (5,249 ESTs), pigeon (4,931 ESTs) and zebra finch (92,142 ESTs). We identified 484 chicken, 48 turkey, 2 duck, 6 pigeon and 82 zebra finch ESTs as potential α-keratins and annotated them using the above method. Of the potential α-keratin ESTs, 219 chicken, 11 turkey and 6 zebra finch ESTs were annotated as α-keratins while none of the duck or pigeon ESTs were annotated as α-keratins.

### Copy number assessment

Species have a shared history that can be represented by a phylogeny and as such, species cannot be considered independent data points in statistical analyses, we therefore employed computer simulations to produce phylogenetic ANOVA p-values in our copy number assessments. As we do not have a species tree that includes the birds, mammals and reptiles used in this study, the statistical analyses of copy number differences for α-keratins were performed by calculating the mean for each group and fold change between the groups.

For the β-keratins, we tested the null hypothesis that the variance of each β-keratin subfamily (claw, feather, scale and keratinocyte) is equal to the variance of the total number of β-keratins using Levene’s test and the SPSS software 21 (IBM Corporation, Armonk, NY).

In order to test the null hypothesis that the proportions of β-keratin subfamilies (claw, feather, scale and keratinocyte β-keratins) are the same for all birds, we utilized standard and phylogenetic ANOVA implemented in the R package GEIGER v 2.0.1 [76,77] and the species tree from Jarvis et al. [42] with 10,000 computer simulations. The proportional data used in these analyses were transformed in SPSS using twice the angle (measured in radians) whose trigonometric sine (Arc sine) equals the square root of the proportion being transformed. Furthermore, the transformed data was tested for normality using the lilliefors (Kolmogorov-Smirnov) test in SPSS and found to be normally distributed. Following phylogenetic ANOVA analyses, the residuals were analyzed and found to be normally distributed with the lilliefors (Kolmogorov-Smirnov) test implemented using the R package nortest.

For the phylogenetic ANOVA analyses, we grouped birds according to their phylogenetic relatedness or lifestyle (aquatic or predatory). For species phylogenetic relatedness, we performed two analyses. The first grouped Paleognathae (ostrich and tinamou) compared to all other birds (Neognathae), the second grouped Paleognathae and Galloanserae (chicken, turkey and duck) compared to all other birds (see Additional file 3; [42]). For the first lifestyle comparison, we grouped the aquatic and semi-aquatic birds (See Additional file 1; [41], Additional file 3; [42]) and compared them to the “land” birds (the terrestrial birds). As the eagles do not cluster with other aquatic birds (Additional file 3; [42]), we also performed the aquatic lifestyle comparison with the eagles grouped with land birds. The final lifestyle comparison analyzed if birds with a predatory lifestyle have any significant differences when compared to the remaining birds (Additional file 1; [41], Additional file 3; [42]).

### Phylogenetic analyses

All phylogenetic analyses were performed using RAxML version 7.4.4 with 1000 bootstrap replicates and the rapid bootstrap analysis [78]. Amino acid substitution models were selected using Prottest version 3.2 [79] and amino acid alignments were performed using ClustalW 2.0.10 [80].

The α-keratin Type I dataset consisted of a total of 580 sequences and the Type II dataset consisted of 625 sequences obtained from the genomes of 48 birds, the American alligator and green sea turtle [43,44], while the sequences used for the green anole and human were downloaded via NCBI (Additional file 6). The β-keratin phylogeny (Additional file 4; [41]) was performed using all 1,623 complete bird genes and the β-keratins from three non-avian reptiles; the green anole lizard [35], green sea turtle [10] and American alligator [9] for a total of 1,698 β-keratins.

Previous molecular analyses of α-keratins indicated that Type I and II sequences should not be analyzed together because the high degree of sequence divergence between the two types result in non-informative phylogenies [7]. Only amino acid sequences containing the entire central helical domain (rod domain) were used in phylogenetic analyses. For the Type II α-keratins this eliminated the 46 KRT6A avian genes.

The Prottest program found that the JTT model with a gamma distribution as the best fit model under the Akaike Information Criterion and Bayesian Information Criterion for both Type I and II α-keratins. Model selection for the β-keratins found that the WAG amino acid model with a gamma distribution as the best fit model under the Akaike Information Criterion and Bayesian Information Criterion for β-keratins.

### Genomic orientation figures

For Type I and II α-keratins, we constructed a proposed genomic orientation based on all of the 48 birds, of which each had at least one genomic locus with at least 2 or more gene variants for both Type I and II α-keratins. Although the results presented in Figures 1B and 2B is representative of most birds, some bird species have additional gene duplications not displayed in the figures. For Type I α-keratins, the red-legged seriema has an additional KRT12 next to the KRT12 on Figure 1B. Type II α-keratins showed more variation. Five birds (MacQueen’s bustard, cuckoo-roller, hoatzin, red-crested turaco and barn owl) have an additional KRT75 between clade C1 and C2 in Figure 2B. Also, four birds (golden-collared manakin, white-tailed tropicbird, downy woodpecker and great-crested grebe) have an additional KRT5 between clade C1 and C2. Two species (Macqueen’s bustard and cuckoo-roller) have an additional cochleal gene in clade C2. Finally, the white-tailed eagle has an additional KRT84 between clade C1 and C2 and the white-tailed tropicbird has an additional KRT73 next to the other KRT73 in clade B1. The annotation of the gene variants is based upon the molecular phylogenies in Figures 1A and 2A.

For the β-keratins, we constructed a genomic alignment of the β-keratin locus containing all of the β-keratin subfamilies (microchromosome 25 of the chicken and zebra finch) for birds with at least one genomic locus with at least 2 β-keratin subfamilies so we could ascertain the orientation of each genomic locus relative to the other birds. A total of 20 phylogenetically diverse species are included in Figure 3, which comprise closely related species pairs (penguins and finches) that illustrate genomic conservation at the fine and broad taxonomic scale across birds. The line breaks for each species is indicative of different genomic scaffolds or contigs. Even though species may lack a region or genes of a β-keratin subfamily (such as the duck and clade 2 claw β-keratins) does not mean they do not have clade 2 claw β-keratins, but that the species does not have a genomic locus with at least 2 subfamilies covering that area. Furthermore, the genes were aligned based on gene orientation and to the species with the highest number of genes similar to DNA sequence alignments with indels. Therefore, species such as the kea and pigeon appear to have a large gap for the scale β-keratins when in fact the genomic region was extended for the chicken which has the highest number of scale β-keratins. Arrows indicate the transcriptional direction. Incomplete β-keratin genes are indicated by white filled arrows, while the solid colored arrows indicate complete genes. The annotation of the β-keratin genes is based upon our annotation (above the figure) and phylogenetic analyses (below the figure).

### Differential α- and β-keratin expression in chicken epidermal tissues during embryogenesis

We investigated the expression profiles of α- and β-keratins during feather and scale development in the chicken using a customized version of the chicken 44 K Agilent microarray [50]. This 60-mer oligonucleotide microarray was validated and tested on a diverse array of tissues and was found to have high sensitivity and specificity [50]. In order to include α-keratins and β-keratins, we removed genes that we deemed not important in this study, such as sensory receptor genes, avian viruses, immune genes and genes related to vision. We were able to create unique 60-mer oligonucleotide sequences for all 27 α-keratins and 102 β-keratins using the online tool, Agilent’s eArray application (Agilent Technologies; Palo Alto, CA).

Macro tissue dissections from the chicken dorsal feather and scutate scale were taken at day 8, 17 and 19 of embryonic development, while the wing feather dissections were taken at day 17 and 19. All tissues, immediately following dissection, were fixed in RNAlater (Qiagen; Germantown, MD). Chicken tissue samples were kindly provided by Richard Goodwin at the University Of South Carolina School Of Medicine in Columbia, SC. RNA extractions were performed using the Qiagen miRNeasy Mini Kit (Qiagen; Germantown, MD). RNA quality and quantity were checked by both the Agilent Technologies 2100 Bioanalyzer (Agilent Technologies; Palo Alto, CA) and Thermo Scientific NanoDrop 2000c spectrometer (Thermo Scientific; Waltham, MA).

RNA was converted to cDNA, labeled and hybridized to the aforementioned single color custom Agilent microarray gene chip at the South Carolina College of Pharmacy Microarray Core Facility (University of South Carolina, Columbia, S.C.). All samples were analyzed in replicates of four, except for day 8 scutate scale which was analyzed in triplicate. The 8 samples were used to perform 14 sample comparisons (See Table 2 and Additional file 5). Both clustering and sample comparisons were performed using Agilent GeneSpring software12.5 (Agilent Technologies; Palo Alto, CA). Sample comparisons were performed using the Mann–Whitney U-test with a p-value cut-off of 0.05 and fold change cut-off of 2.0.

### Availability of supporting data

The data sets supporting the results of this study can be found at Beijing Genomics Institute (http://phybirds.genomics.org.cn) and the GigaScience Genome Downloads (http://gigadb.org/dataset/101000) [41,42].

## Additional files

Additional file 1:
**Is reprinted with permission from Zhang** ***et al*** **. [** 41 **] and lists the copy numbers of the Alpha (α)- and beta (β)-keratin for each species of mammal, reptile and bird used in this study.** Copy numbers for mammalian α-keratins [7], green anole β-keratins [35], green sea turtle β-keratins [10] and crocodilian β-keratins [9] were obtained from their respective studies. Species in bold have an aquatic or semi-aquatic lifestyle and those with an asterisk next to their name have a predatory lifestyle. Additional file 2:
**Contains tables listing the number of α- and β-keratins determined by our annotation and phylogenies for each species in this study.**
Additional file 3:
**Contains the 48 bird genome scale phylogeny and is reprinted with permission from Jarvis** ***et al.*** **[** 42 **].**
Additional file 4:
**Is reprinted and modified with permission from Zhang** ***et al*** **. [** 41 **] and illustrates the molecular phylogeny of β-keratins.** This figure displays the maximum likelihood phylogeny of β-keratins from the green anole lizard, green sea turtle, American alligator and the 48 birds. The green anole lizard β-keratins formed a significant clade and were subsequently used as an outgroup. Colored clades are statistically significant except for the scale β-keratins, which only have a subset forming a significant clade. The green sea turtle β-keratin genes are found in the keratinocyte and claw β-keratin clades while the American alligator β-keratin genes are found in the keratinocyte, scale and claw β-keratin clades. The feather β-keratin clade is composed of only avian β-keratins. The feather β-keratin clade is further annotated based on genomic loci (chromosomes) of the chicken and/or zebra finch feather β-keratins. Chr. is an abbreviation for chromosome and F-L indicates feather-like β-keratins. Chr. 27 A corresponds to genes found on the 5′ most array of feather β-keratins on zebra finch microchromosome 27 (see Figure 3 of Greenwold and Sawyer [8]) and related genes, while Chr. 27 B are the genes on the 3′ most array of feather β-keratins on zebra finch microchromosome 27 and chicken chromosome 5 and microchromosome 27. Additional file 5:
**Contains tables listing the differentially expressed genes and their p-value, fold change and regulation for each sample comparison.**
Additional file 6:
**Contains tables listing species and identification number for the sequences used as queries in genome searches.**
