# Supplementary figures and images for: Dynamic evolution of the alpha (α) and beta (β) keratins has accompanied integument diversification and the adaptation of birds into novel lifestyles
Source: BMC Evol Biol. 2014 Dec 12;14:249. doi: 10.1186/s12862-014-0249-1 (PMC4264316; doi:10.1186/s12862-014-0249-1)

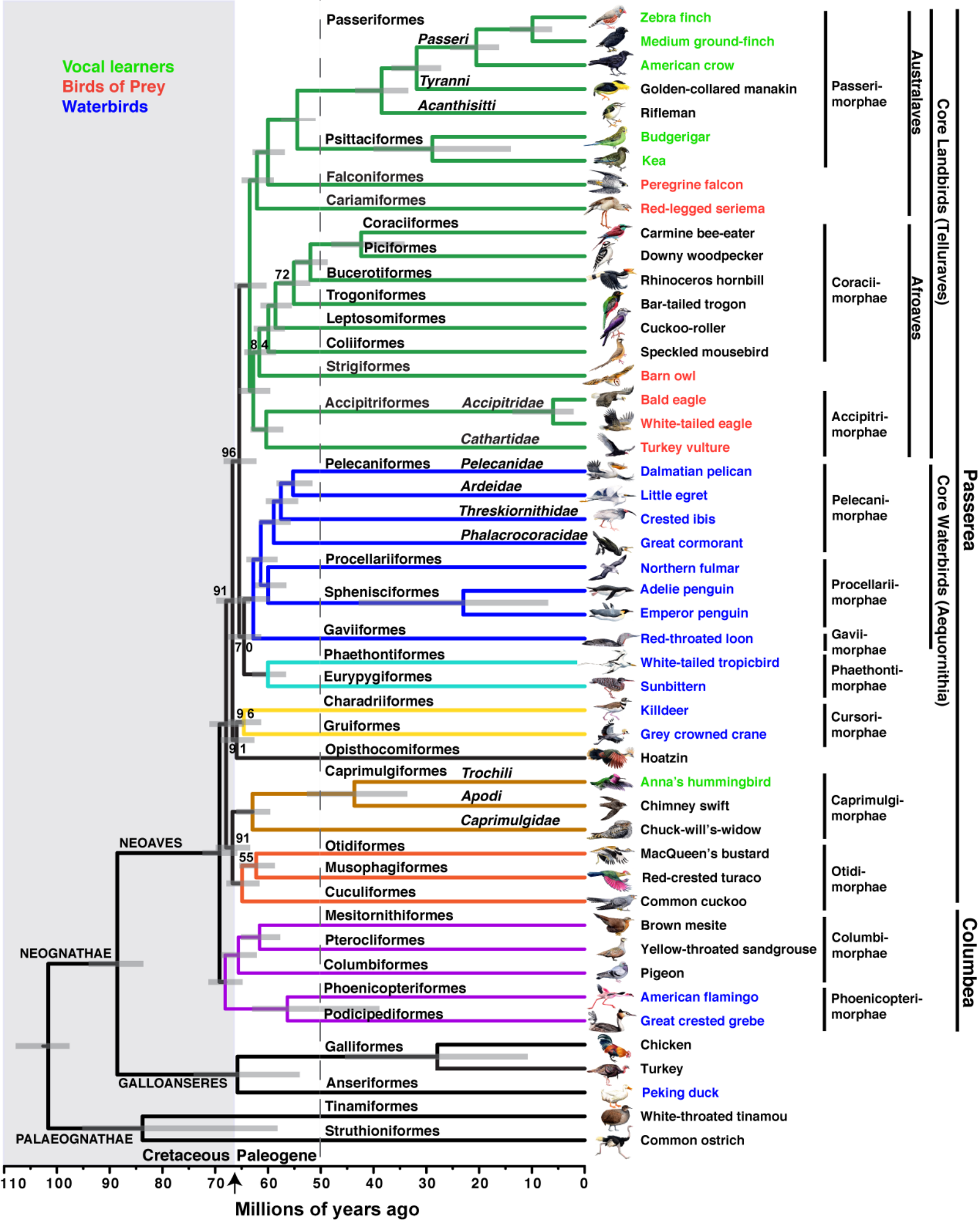

Supplement: Additional file 3: — Contains the 48 bird genome scale phylogeny and is reprinted with permission from Jarvis et al. [ 42 ]. [file 12862_2014_249_MOESM3_ESM.pdf]

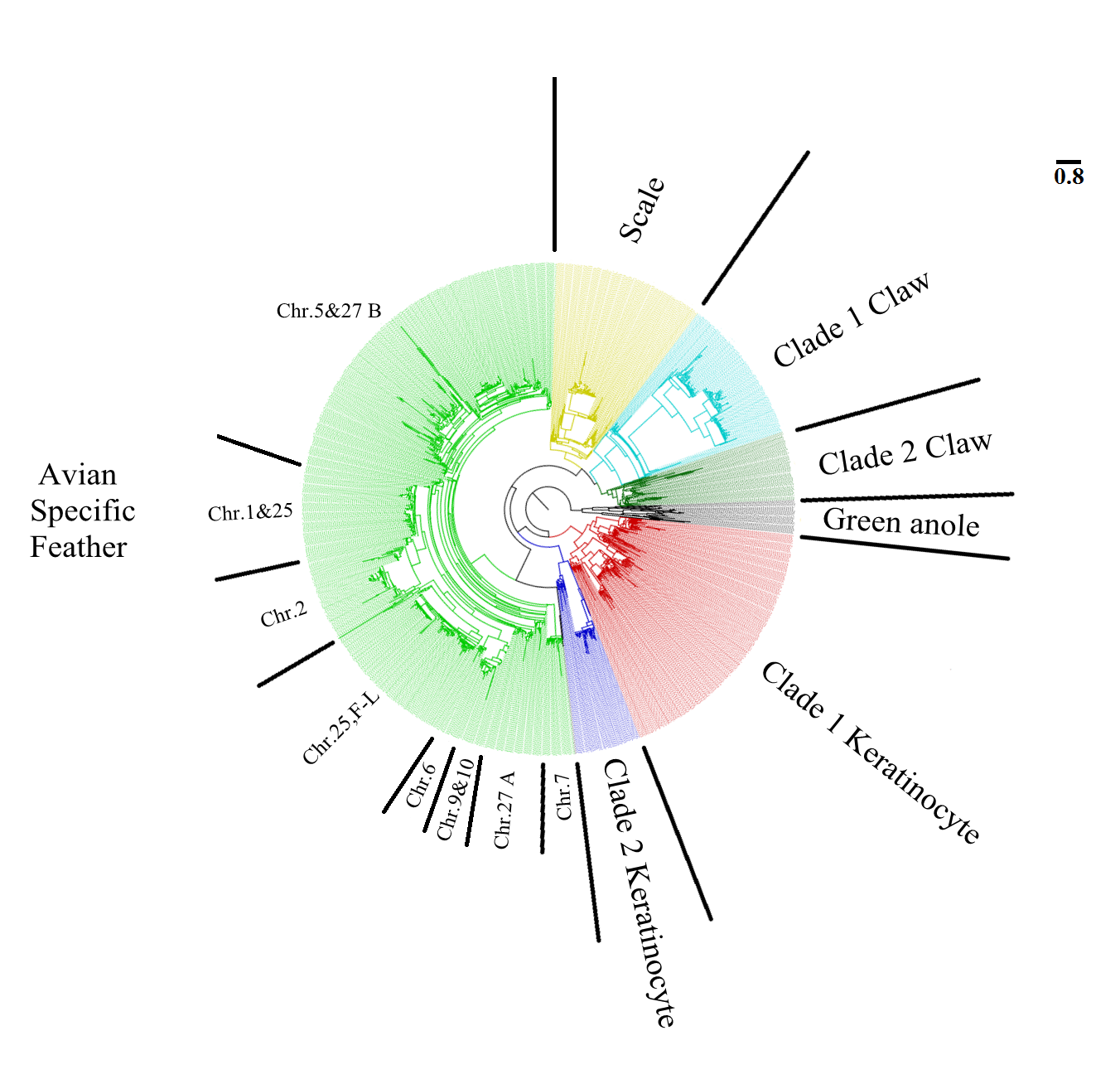

Supplement: Additional file 4: — Is reprinted and modified with permission from Zhang et al . [ 41 ] and illustrates the molecular phylogeny of β-keratins. This figure displays the maximum likelihood phylogeny of β-keratins from the green anole lizard, green sea turtle, American alligator and the 48 birds. The green anole lizard β-keratins formed a significant clade and were subsequently used as an outgroup. Colored clades are statistically significant except for the scale β-keratins, which only have a subset forming a significant clade. The green sea turtle β-keratin genes are found in the keratinocyte and claw β-keratin clades while the American alligator β-keratin genes are found in the keratinocyte, scale and claw β-keratin clades. The feather β-keratin clade is composed of only avian β-keratins. The feather β-keratin clade is further annotated based on genomic loci (chromosomes) of the chicken and/or zebra finch feather β-keratins. Chr. is an abbreviation for chromosome and F-L indicates feather-like β-keratins. Chr. 27 A corresponds to genes found on the 5′ most array of feather β-keratins on zebra finch microchromosome 27 (see Figure 3 of Greenwold and Sawyer [8]) and related genes, while Chr. 27 B are the genes on the 3′ most array of feather β-keratins on zebra finch microchromosome 27 and chicken chromosome 5 and microchromosome 27. [file 12862_2014_249_MOESM4_ESM.png]
